# Visual Search of Neuropil-Enriched RNAs from Brain In Situ Hybridization Data through the Image Analysis Pipeline Hippo-ATESC

**DOI:** 10.1371/journal.pone.0074481

**Published:** 2013-09-09

**Authors:** Roberto Ugolotti, Pablo Mesejo, Samantha Zongaro, Barbara Bardoni, Gaia Berto, Federico Bianchi, Ivan Molineris, Mario Giacobini, Stefano Cagnoni, Ferdinando Di Cunto

**Affiliations:** 1 Department of Information Engineering, University of Parma, Parma, Italy; 2 CNRS UMR 7275, Institute of Molecular and Cellular Pharmacology, University of Nice-Sophia Antipolis, Valbonne, France; 3 Molecular Biotechnology Center, Department of Molecular Biotechnology and Health Sciences, University of Torino, Torino, Italy; 4 Department of Veterinary Sciences, University of Torino, Torino, Italy; 5 Neuroscience Institute of Torino (NIT), University of Torino, Torino, Italy; University of Illinois-Chicago, United States of America

## Abstract

**Motivation:**

RNA molecules specifically enriched in the neuropil of neuronal cells and in particular in dendritic spines are of great interest for neurobiology in virtue of their involvement in synaptic structure and plasticity. The systematic recognition of such molecules is therefore a very important task. High resolution images of RNA *in situ* hybridization experiments contained in the Allen Brain Atlas (ABA) represent a very rich resource to identify them and have been so far exploited for this task through human-expert analysis. However, software tools that may automatically address the same objective are not very well developed.

**Results:**

In this study we describe an automatic method for exploring in situ hybridization data and discover neuropil-enriched RNAs in the mouse hippocampus. We called it Hippo-ATESC (Automatic Texture Extraction from the Hippocampal region using Soft Computing). Bioinformatic validation showed that the Hippo-ATESC is very efficient in the recognition of RNAs which are manually identified by expert curators as neuropil-enriched on the same image series. Moreover, we show that our method can also highlight genes revealed by microdissection-based methods but missed by human visual inspection. We experimentally validated our approach by identifying a non-coding transcript enriched in mouse synaptosomes. The code is freely available on the web at http://ibislab.ce.unipr.it/software/hippo/.

## Introduction

The communication between neuronal cells is primarily achieved through chemical transmission at synapses, specialized subcellular structures in which axons and dendrites of connected neurons are closely juxtaposed. In the case of excitatory contacts, the most intensely studied and better understood type, synapses are formed at structures known as dendritic spines (DS), small protrusions of the dendritic membrane that compartmentalize the biochemical events activated by synaptic transmission [1], [2]. One of the most remarkable features of DS is that their shape and efficiency can be individually modified as a function of activity [3]. Targeting of coding and non-coding RNAs to axons, dendrites and to dendritic spines (collectively referred to as the neuropil) plays a very important role in the localized control of gene expression underlying these phenomena [4]–[6]. Among the protein-coding RNAs, Ca2+-calmodulin-dependent protein kinase alpha subunit (Camk2a) [7], [8], Map2 [9], Shank [10], β-actin [11] and Arc [12] are the best documented examples of neuropil-enriched mRNAs. On the other hand, the dendrite enriched non-coding transcript Bc1 has been shown to regulate synaptic plasticity by locally repressing the translational of mGluR receptors [13]. Therefore, the automatic detection of neuropil-enriched transcripts has become a very important issue. This problem has been so far addressed through two main general strategies: the direct measurement of RNA molecules in extracts prepared from microdissected neuropil or the use of RNA in situ hybridization on cells and tissues. The rodent hippocampus has been fruitfully used for microdissection-based expression studies, in virtue of the very precise arrangement of cell bodies and neuronal projection that characterize this structure [14], [15]. In combination with microarray-based measurements, this technique has led to the identification of approximately 200 transcripts enriched in the neuropil, as compared to cell bodies [14]–[16]. More recently, an RNA-seq-based study has shown that the hippocampal neuropil contains approximately 2550 coding transcripts [16]. However, such a study did not provide information about the relative enrichment of transcripts between the neuropil and the cell bodies and did not report any information about the non-coding transcripts.

Systematic in situ hybridizations on adult mouse brain with probes derived from virtually all protein coding genes and from many non-coding transcripts have been performed within the Allen Brain Atlas project (ABA) [17], [18]. The analyses of ABA images so far performed have identified many neuropil-enriched transcripts [17], but suffer of two main limitations. On the one hand, although the resolution limit of the ABA images is 1.07 µm, theoretically allowing for the discrimination of sub-cellular details, their systematic mining by automatic tools has been focused on exploring the general gene expression patterns at low resolution (200 µm), or on measuring expression in cell bodies [17]. On the other hand, human expert inspection of high-resolution images has led to the highly specific identification of 57 dendrite-enriched RNAs, but may have significantly underestimated the number of neuropil-enriched RNAs, as it would seem if considering the much higher number of neuropil transcripts detected by RNA-seq [16].

In this study we describe the implementation of an automatic pipeline aiming at detecting transcripts enriched in the hippocampal neuropil of adult mice, by systematically exploring the high resolution images contained in the ABA. The method is based on the automatic identification of the different hippocampal sub-regions in high resolution ABA images, followed by the extraction of analysis of many different image-texture features. On this basis, we ranked the mouse coding and non-coding transcripts represented within the ABA according to their similarity to well known neuropil-enriched transcripts. The comparison of our ranking with the results of microdissection studies confirmed the high specificity of our method. We experimentally validated our results by identifying a new non-coding transcript associated to the synaptodendrosomal compartment.

## Methods

### The Hippo-ATESC Pipeline

**Figure 1:**
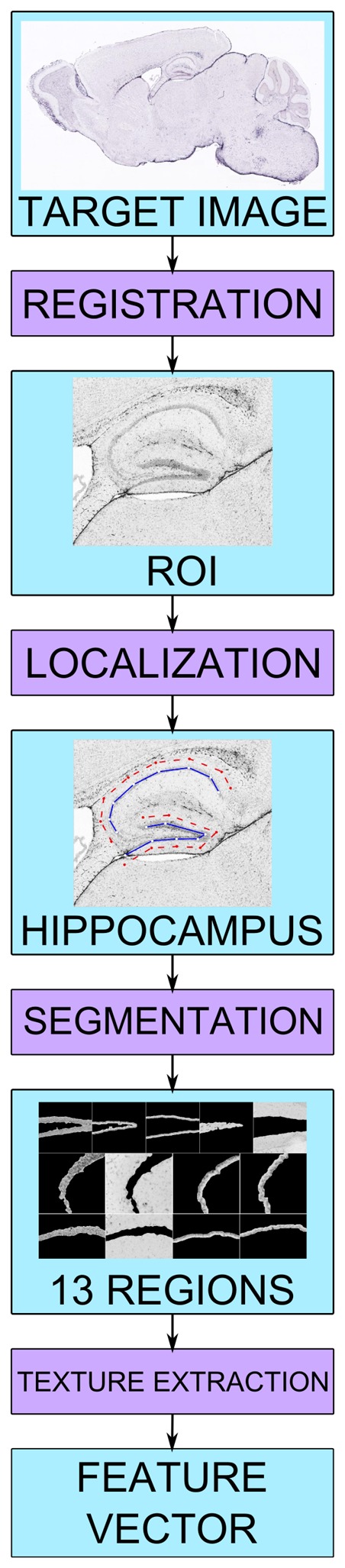
Schematic representation of the Hippo-ATESC pipeline.

**Figure 2:**
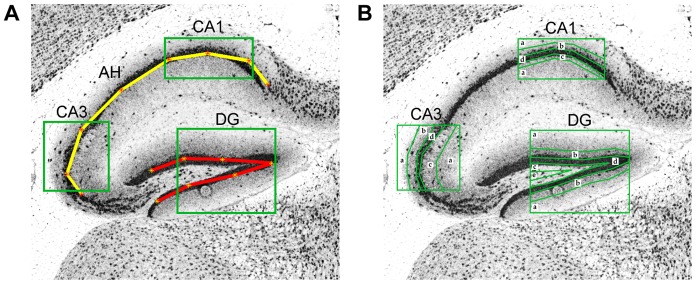
Graphical illustration of the localization and segmentation steps. (A) The deformable model of the Ammon’s horn (AH) and of the dentate gyrus (DG) are represented by a yellow line connecting red stars and by a red line connecting yellow stars, respectively. Selection of the regions of interest is represented by green boxes. (B) The regions described in Table 1 are indicated.

The automatic pipeline is based on three main steps: i) localization of relevant hippocampal sub-regions; ii) characterization of the texture of these regions; iii) training of a model for neuropil-enriched transcripts, on the basis of prototype mRNAs. A schematic representation of the procedure is given in Fig. 1. In order to identify the main hippocampal regions within ABA *in situ* hybridization images, we adapted an automatic method that we previously described [19]. In this method, the localization of hippocampal main structures, i.e. the Ammon’s Horne (AH) and the dentate gyrus (DG) is achieved by searching the parameters of an empirically-derived deformable model (DM) [20], which maximizes its overlap with the corresponding anatomical structure in the brain image using a metaheuristic [21] and gives as output the location of the hippocampal region achieving a good trade-off between accuracy and speed [19] (Fig. 2A). Afterwards, we performed a segmentation step in order to identify 13 different hippocampal sub-regions, corresponding to specific sub-sections of the dentate gyrus and of the CA1 and CA3 regions of the Ammon’s Horn (Fig. 2B). From these regions, we extracted 220 textural features from each image. From this large dataset we first extracted a training set composed of three prototypical neuropil-enriched genes and 17 negative examples, based on which a genetic algorithm [22] selected a subset of 52 significant features (Table S1). Finally, for every gene, we extracted its 52-feature vector and compared it to a reference vector (the average of the three prototype vectors) using the Pearson coefficient as a distance measure [23].

### Localization and Segmentation of Hippocampal AH and DG

The method used to localize the AH and the DG of the hippocampus within ABA sagittal sections is described in more details in [19]. Briefly, it can be divided into two stages: (i) selection of the corresponding slice in the reference atlas based on a two-step affine registration process, and (ii) proper localization of the hippocampus. The first stage recognizes the location, within the brain, of the section displayed in the target image based on a comparison with the images of a reference atlas, and extracts the region of interest (ROI) where the hippocampus is most likely to be located. The following stage performs the proper localization of the anatomical structure under study using statistical shape models. In particular, before localization, the ROI that has been extracted is preprocessed to remove noise and refine the detection of the background. Consequently, an intensity normalization process using a training set of ROIs, followed by a contrast-limited adaptive histogram equalization, and the saturation of the darkest/brightest parts in the ROI were used. After that, a binarization process using Otsùs thresholding method before keeping the five biggest connected components was applied. After this preliminary step, a DM inspired by Active Shape Models (ASM) [20] was employed to precisely localize the hippocampus, using a training set of shapes extracted from five to twelve images for each reference slice in the atlas, and deriving a model with eight reference points for the AH and seven points for the DG. This ASM approach is based on an energy minimization framework optimized using a metaheuristic called Differential Evolution (DE) [24]. To fully specify the algorithm, we used a crossover rate of 0.9, while the scalar F was set to 0.7. Uniform crossover and DE/target-to-best/1 mutation were employed over a population of 64 individuals and 200 generations. During the registration phase, a classical gradient-based local search method [25] was used in the first step, while Particle Swarm Optimization (PSO) [26] was applied in the second one. Our PSO implementation was run with 24 particles, 40 iterations, c1 = c2 = 2.05, and an inertia factor linearly decreasing with time from 1.0 to 0.1.

The control points of the DM were used to locate different areas of the hippocampus constantly containing both cell bodies and neuropil. They were approximately centered on the region of maximum curvature of the CA1 region and of the CA3 region in the stratum pyramidale of the AH, and in the medial half of the stratum granulosum (sg) for what concerns the DG (Fig. 2A). Afterwards, these areas were first segmented using a system based on Otsu's thresholding method [27], and then further subdivided to obtain 13 different regions of interest (Fig. 2B), able to describe most of the variability of hippocampus' visual features. The 13 regions were then checked to automatically reject incorrect segmentations as follows. A Random Forest (RF) [28] was trained to distinguish between the points lying inside Stratum Pyramidale (sp) and Stratum Granulosum (sg) and those lying outside. Twelve random points were selected in the regions identified in Fig. 1B as CA1 a, CA1 d, CA3 a, CA3 d, DG a, DG d, and classified using the trained RF. Then, the percentage of points correctly classified, along with some statistics of the images containing the regions (area, standard deviation) were processed by another RF, to finally evaluate the segmentation process. If the result was considered unreliable and had to be discarded, the entire process was repeated selecting a different section of the same series from the ABA, and if all slices within the brain region in which the hippocampus is clearly visible had been unsuccessfully processed, the gene was finally discarded. Over 9510 series which were tested, only 255 of were discarded (2.68%), including the ones dismissed due to errors in the ABA, e.g. ruined or missing images. Genes with multiple probes or multiple image series available in the ABA were treated independently because different probes could reveal different transcripts, characterized by dissimilar expression profiles.

### Texture Extraction

Textural features of the first and second order were extracted by each region. The size of the windows for each region, as well as the textural features used, are reported in Table 1. The total number of features, i.e. the size of the vector which encodes the visual characteristics of a gene is therefore 220. These features are both of the first order (obtained directly from the image) and of the second order (obtained using Gray Level Co-occurrence Matrix). The Gray Level Co-occurrence Matrix (GLCM) [29] is a well-established method to represent textural information of an image I by defining the distribution of co-occurrence values:
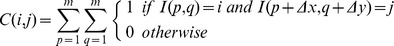
where m represents the size of the window to analyze, i and j represent the intensity levels of image I (usually the number of levels is reduced to avoid unfeasible sizes of the matrix: in this work we set this number to 16). The offset (Δx, Δy) represents the distance in pixel and the direction between two points. For instance, one can be interested only in vertical or horizontal patterns. In this work, we consider symmetric and non-directional relations between points. Once the matrix is computed, it can be used to extract indices that represent particular features of the image like, for instance, its contrast or its homogeneity.

**Table 1:** Different features used for the ranking.

CA1a	CA1b	CA1c	CA1d	CA3a	CA3b	CA3c	CA3d	DGa	DGb	DGc	DGd	DGe
18,36	24	24	14,28	18,36	24	24	14,28	18,36	24	24	14,28	18,36
First Order		Mean, Standard Deviation, Coefficient of Variation, Skewness, Kurtosis, Energy, Entropy										
Second Order		Contrast, Correlation, Energy, Homogeneity										

The first row contains the 13 regions under consideration (Fig. 2). The second row shows the window sizes used to compute textural features in each region; for example, “18, 36″ means two different sizes (of 18×18 and 36×36 pixels, respectively). Each feature is the average of the result obtained in windows that cover, as much as possible, the segmented region.

### Model Training and Ranking Procedure

A small dataset composed of 20 genes was used to train the system. The idea was to select a feature subset able both to represent well the dendrite-enriched mRNAs and to successfully distinguish them from negative examples. To do so, we used as positive examples (prototypes) three genes which are well known for the dendrite and spine enrichment of their mRNA, namely Camk2a (ID = 12322) [7], [8], Map2 (ID = 17756) [9] and Arc (ID = 11838) [12]. Moreover, we used as negative examples 17 genes with different characteristics. In particular, we chose: Camk2b (ID = 12323), Tubb3 (ID = 22152) and Grin1 (ID = 14810), i.e. three genes whose mRNA is specifically expressed in neurons but is not transported in dendrites; Gapdh (ID = 14433), Pgk1 (ID = 18655) and Pfkm (ID = 18642), i.e. three ubiquitous metabolic enzymes whose mRNA is strongly expressed in neurons but is not transported in dendrites; Gfap (ID = 14580) and Slc1A2 (ID = 20511), i.e. two genes expressed glial cells, particularly in astrocytes; Mag (ID = 17136), Mog (ID = 17441) and Mbp (ID = 17196), i.e. three genes genes expressed in oligodendrocytes; Gad1 (ID = 14415) and Slc6A1 (ID = 232333), i.e. two genes expressed in GABA-ergic interneurons; Slc1A1 (ID = 20510), Slc1A3 (ID = 20512) and Slc17A7 (ID = 72961), i.e. three genes genes expressed in glutamatergic neurons; glial cells, particularly in astrocytes; Sox2ot (320478), a gene producing a non-coding RNA localized in the nucleus. The images chosen for the above genes were reviewed by an expert, who confirmed the correspondence of the expected expression pattern with the pattern revealed by the ABA in situ hybridizations. Features that, in this small dataset, showed a very high correlation between each other (>0.99) were considered as being the same, and one of them was removed. After this, a binary genetic algorithm (with population size 50, run for 300 generations, with crossover rate of 0.8, mutation rate 0.06, and tournament selection of size 4) was used. Every individual in the population encodes a feature subset and the silhouette index [30] is used to evaluate its effectiveness. This index is computed using the selected subset of features and considering the positive examples in one group and the negative examples in another.

The silhouette index is a measure of an object’s similarity to the others of the same group and dissimilarity from the elements in the other groups. In the following formula, “a” identifies the average distance (or dissimilarity) between an element with the data in its own group, while “b” is the average dissimilarity between the element and the elements of the other group.
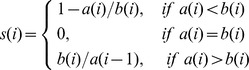



The rationale underlying the use of a GA to solve a search space in which, with 220 features, there are 2∧220 possible feature subsets, can be found in the ability of such algorithms to solve NP-complete problems [31].

Since a genetic algorithm is a stochastic metaheuristic, which may produce different solutions each time it runs, this procedure was repeated 15 times and the features selected in at least 50% of the runs were chosen. This led us to select a subset of 52 features (see Table S1), which were used to process all genes in the ABA and compute the Pearson coefficient between their feature vector and the prototype one, generated by averaging the features of three prototype genes. The results using the 220 features were 0.5066, 0.4302, 0.8150, while using only the selected features were 0.8610, 0.8796, 0.9441; proving the ability of the proposed feature-selection method to facilitate the detection of correlations between genes. It is important to underscore that this pipeline has general applicability and would be able to rank genes according to their similarity to any kind of features, only by providing it a training set including positive and negative examples.

### Candidates Validation by qPCR

Total RNA was extracted from brains of adult C57BL/6J mice. All animals were handled in accordance with good animal practice as defined by the relevant national animal welfare bodies, equivalent to the European Convention for the Protection of Vertebrate Animals used for Experimental and other Scientific Purposes (ETS 123). Mouse experimentation protocols were approved by the Nice Sophia Antipolis University regional animal safety committee (CIEPAL-Azur). RNA preparation from total brain and crude synaptosomes was performed as previously described [32]. For each sample 500 ng of RNA were retrotranscribed with SuperscriptIII (invitrogen) according to the manufacturer's instruction using both random primers and OligodT. The quantitative real-time PCR was performed on a LightCycler 480 Real-Time PCR System (Roche), using the qPCR Core kit for SYBR Green (Eurogentec), according to the manufacturer’s instruction. For each reaction 6.25 ng of cDNA were used. The amplification protocol was: 95°C for 10′, followed by 45 cycles of 95°C for 10′′, 60°C for 45′′ and 72°C for 10′′. The relative expression of the transcripts was quantified with the 2*^−^* ^ΔCt^ method [33] using FMR1 as a reference. For each synaptosomal or brain sample, ΔCt is the result of the subtraction of the Ct values obtained for Fmr1 (used as a reference, since it is present at equal levels in both synaptosomes and brain) from the Ct values of the non-coding RNAs. This method allows to compare the relative expression levels of a set of transcripts. In all qPCRs a positive control, 2900097C17Rik, was always present. The enrichment of non-coding RNA in the synaptosomal preparation was expressed as the ratio of 2*^−^* ^ΔCt^ between synaptosomal and total brain RNA.

The primers used are: 8030498B09Rik = Forward ATTGGGTACATGCTCAGGACA, Reverse AGCCAGGGCTACACAGAGAA; LOC433089 = Forward ATGACCATGGCCTTTTCATC, Reverse GCTGTGGGGTACAGGGATAA; A830039N20Rik = Forward CATATCACCCCCGTTGTACC, Reverse TTTTCACTTGGCCAAAAAGC; 2700046G09Rik = Forward CTTGTCCTCTCCTGCACCTC, Reverse AAATAACCAGCGGGGCTACT; LOC435897 = Forward ATTCCACGTGATTGGCAACT, Reverse AAATAACCAGCGGGGCTACT; TC1430156 = Forward TGTCACGGTCAGCTCTGTTC, Reverse AGGGTGGGTCTTCAATTCG; 2900097C17Rik = Forward GACAACGGCCATGTAGTGTG, Reverse ATCCTATCCCCAAGCCATTT.

## Results and Discussion

### Detection of Neuropil-enriched Transcripts by the Hippo-ATESC Pipeline

**Figure 3:**
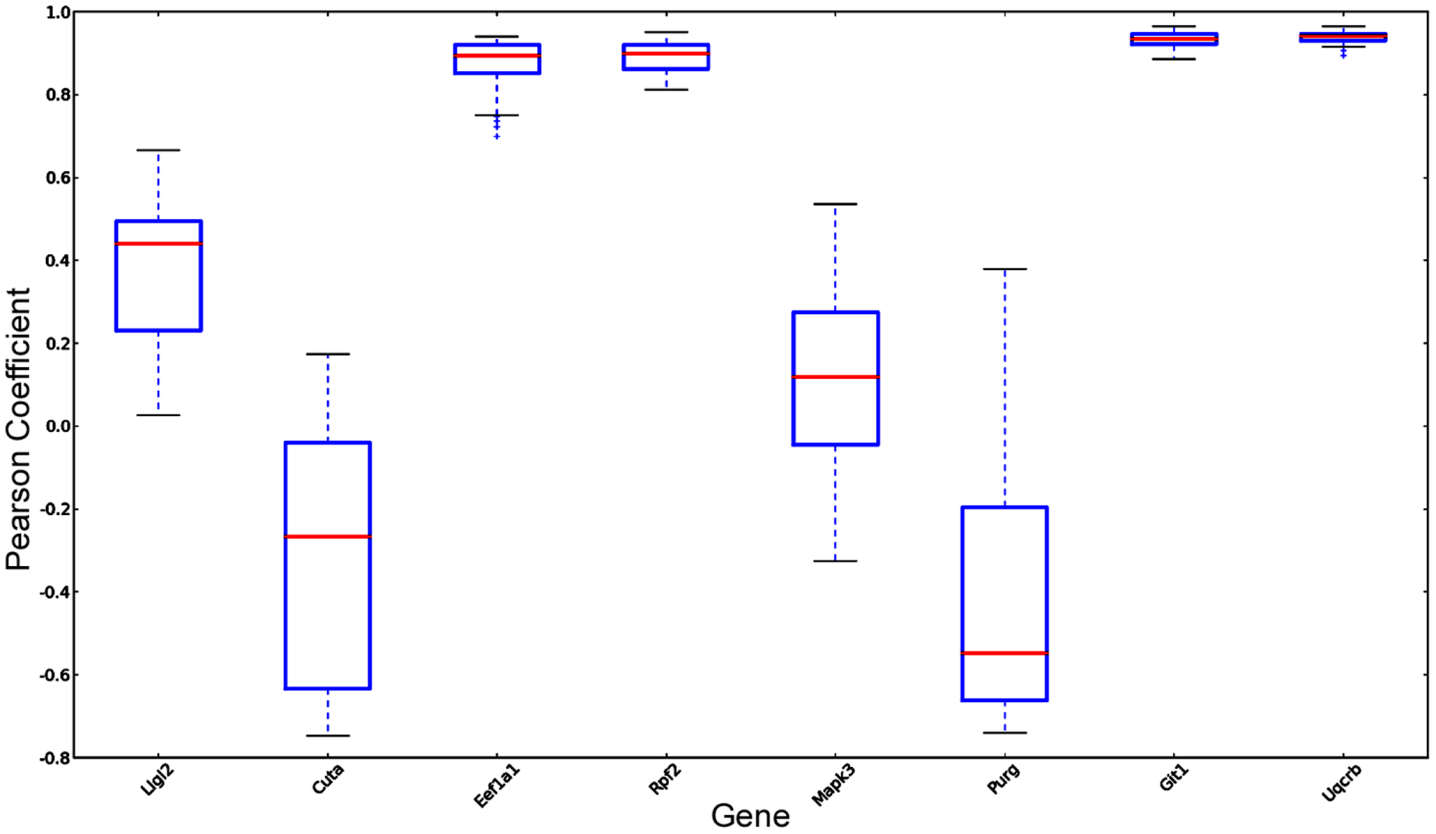
Boxplot of the correlation values of the feature vectors obtained from 6 slices of 8 randomly selected genes, as compared to the prototype vector. Note the very stable behavior of genes characterized by a high Pearson coefficient.

**Figure 4:**
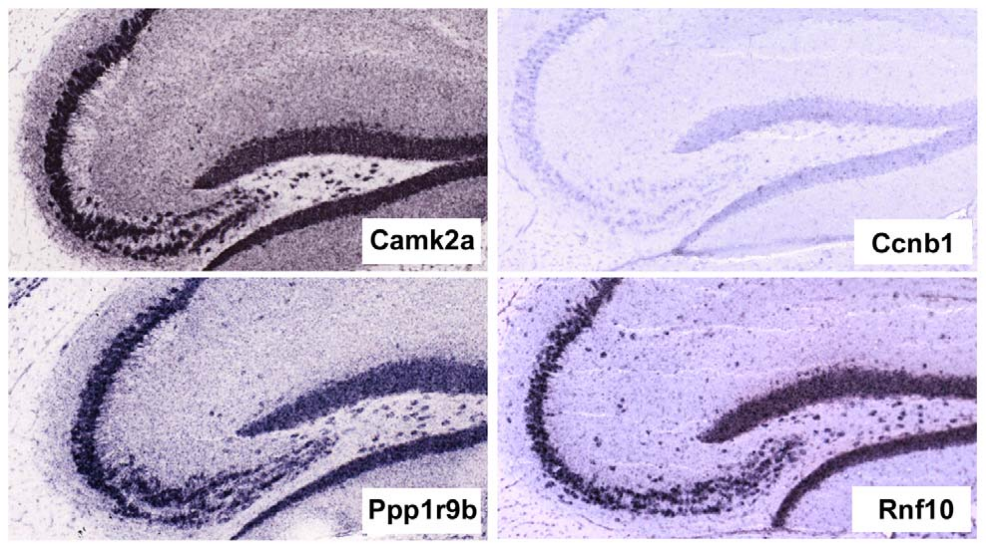
In situ hybridization pattern of the indicated protein-coding genes, obtained from the Allen Brain Atlas. Camk2a was included as a positive control while Ccnb1, which encodes for transcripts expressed only in mitotic cells, was included as a negative control to give an idea of background signal levels.

The Hippo-ATESC pipeline was trained using as positive cases three of the best experimentally characterized neuropil-enriched genes described in the literature (Camk2a, Arc and Map2) as well as 17 negative genes (see the methods section for the details). We then set out to scan parasagittal images contained in the ABA to obtain similarity scores between the texture vectors of all informative probes and a prototype, or reference vector, obtained by averaging the vectors of the prototype genes. In particular, to avoid scoring genes expressed at negligible levels in adult mouse hippocampus, we only considered image series in which the expression level or the expression density reported for the hippocampal region or for the hippocampal formation was above 20, a background level determined on the basis of cell cycle genes which are known to be silent in brain after the end of development. For all the genes that resulted above this threshold (n = 9159), we selected parasagittal sections corresponding to levels from 117 to 175 of the reference atlas. In particular, we selected as default the level 145, which we consider as ‘center’ of the hemisphere, and moved towards the two boundaries if we were not able to get results with the selected slice. For each section we identified the different hippocampal regions and determined the values for the corresponding texture parameters, obtaining vectors of texture features. We then calculated the Pearson correlation coefficients (r) of these vectors with the reference vector. As expected, vectors obtained from sections corresponding to slightly different levels were very similar, as the average Pearson for all the sections of the prototype genes included in the range was equal to 0.88±0.17. Of notice, visual inspection of all these sections revealed that the wide range of r-values obtained with the same probe are caused by specific qualitative problems of some sections, which results in very low scores and would therefore produce false negative results, if the procedure should be employed for classification purposes. Nevertheless, genes showing a high Pearson coefficient to the prototype vector displayed a very stable behavior in this test (some examples are shown in Fig. 3). On this basis, we obtained feature vectors for the default level of all ABA probes above threshold and calculated their r values with respect to the reference vector. Interestingly, the distribution of r values did not show inflection points, indicating that it could be naturally used for classification for evidence-based ranking. To evaluate if such an approach could provide valuable new hypothesis for experimental validation, we first analyzed the 20 top scoring probes corresponding to protein-coding genes (Table 2). As expected, a Gene Ontology analysis performed with the DAVID software [34] revealed that the most significant common keyword associated to the corresponding genes is “dendrite” (P = 0,004). In particular, manual inspection revealed that this list contains some of the best known examples of transcripts localized to dendrites and/or associated with dendritic functions. Indeed, besides to a Camk2a probe not included in the training set, the list contained probes from Dendrin (Dnd) [35], [36], Psd (also known as Efa6a) [37], microtubule-associated protein 2 (Map2) [9], Git1 [38] and Spinophilin (Ppp1r9b) [39], [40]. Accordingly, visual inspection of the corresponding ABA images confirmed a significant signal enrichment in neuropil for many of the probes included in the list Table 2, as it is the case for the Rnf10 gene (Fig. 4). It must be noticed that 7 out the 20 genes are associated to mitochondrial function (Table 2). However, this result is most likely non specific, because it could be easily explained by the high abundance of mitochondria in dendrites. Indeed, the mRNAs of genes mapping to the mitochondrial genome are transcribed within mitochondria, while the mRNAs of nuclear genes encoding mitochondrial oxidative phosphorylation proteins have been found to localize near mitochondria [41]. Therefore, in all these cases a neuropil-enriched pattern would be expected. To obtain a more systematic validation, we concentrated on a group of 257 protein-coding gene-derived probes, characterize by a r value of 0.8 or higher. These probes (Table S2), derived from 117 nuclear genes and from 9 mitochondrial genes, corresponded to 2.7% of all the probes providing in situ hybridization signals significantly higher than background levels. Analysis of the functional annotations of these genes revealed a significant enrichment for phosphoproteins (n = 116, hypergeometric P-value (P) = 2.8E-09) and for GTP-binding proteins (n = 15, P = 4.1E-05), as well as a slight overrepresentation of mitochondrial proteins (n = 30 P = 1.9 E-03). The latter result was even more pronounced if one considers the significant enrichment for probes directly derived from the mitochondrial genome. Besides, it must be considered that other types of “false positive” results may be contained in the top-scoring list, such as the Plp1 transcript, which is well known to be expressed by oligodendrocytes [42].

**Table 2:** List of the top-scoring 20 protein-coding genes defined by the pipeline.

Gene Name	ABA	Entrezgene ID	Pearson	SF	MF
Ddn	71212512	13199	0.964	X	
9530085C10Rik*	73906215	N/A	0.961		X
Camk2a	79360274	12322	0.960	X	
0610005I03Rik*	74357749	N/A	0.957		X
Psd	69352896	73728	0.954	X	
Uqcrb	293241	67530	0.954		X
Mtap2	69549641	17756	0.954	X	
0610009I12Rik*	74357773	N/A	0.953		X
0610006F12Rik*	74357761	N/A	0.950		X
Git1	69672880	216963	0.948	X	
Slc25a23	71280844	66972	0.946		X
Rnf10	294052	50849	0.945		
Muc10	72737	17830	0.943		
Rpl23	70813131	65019	0.939		
7420498E04Rik*	73927706	N/A	0.938		X
Senp2	227749	75826	0.936		
Bcap29	72739	12033	0.932		
Mt1	67767450	17748	0.930		
Ppp1r9b	68151446	217124	0.930	X	
Tubb2a	69838608	22151	0.928		

Gene names marked by a star correspond to genes mapping to the mitochondrial genome. ABA indicates the Allen Brain Atlas experiment number. In the SF and MF columns, the genes which were previously associated to dendritic spine or synaptic function and the genes associated to mitochondrial function are marked, respectively.

To evaluate whether the mRNA produced by these genes could be actually enriched in dendrites, we compared our top ranking list with the list of 2250 coding genes that should compose the mouse hippocampal neuropil, according to recent RNA-seq studies [16]. This comparison revealed 56 common genes, representing a highly significant intersection (Table S2, P = 2E-07). More importantly, we compared our list with a list of 57 transcript manually annotated by experts as dendritically enriched in the original ABA publication [17]. Interestingly, this analysis revealed 12 common genes (P = 2E-17), most of which were concentrated in the top ranks of our list. In comparison, the lists of neuropil-enriched transcripts reported in previous micro-dissection-based studies [14], [15] showed a much less significant intersection with the same ‘ground truth, because each had in common with it only 2 genes, over a total of 170 and 154, respectively (P-value not significant in both cases). In addition, our list had a significant intersection with those resulting from microdissection studies, having 6 genes in common with the list reported in [15] (P-value = 0.009) and 9 genes in common with the list reported in [14] (n = 154, P = 5E-05). The above numbers are in the same order of magnitude of the intersection between the two latter studies (6 common genes, P = 0.0082). Taken together, these results indicate not only that the Hippo-ATESC pipeline can efficiently score neuropil-enriched transcripts that would be identified by human experts, but also that it can highlight neuropil-enriched transcript which escape visual expert analysis but can be revealed by quantitative methods.

### Experimental Validation of Neuropil-enriched Non-coding Transcripts Characterized by High Hippo-ATESC Score

**Figure 5:**
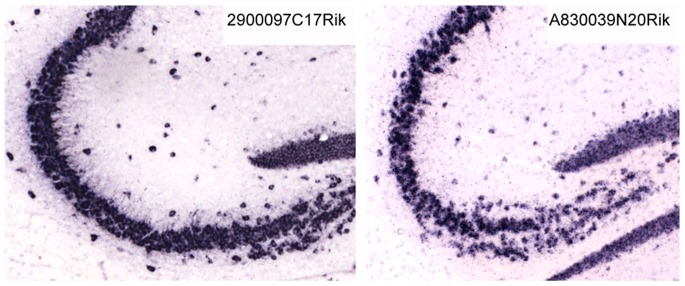
In situ hybridization pattern of the indicated non-coding RNA genes, obtained from the Allen Brain Atlas. For positive and negative control, see Fig. 4.

Considering the good results obtained with protein-coding genes, we would expect that the Hippo-ATESC pipeline should be as effective in highlighting neuropil-enriched non-coding RNAs. Therefore, we analyzed the ranking of probes annotated in the ABA as belonging to non-coding genes. The number of probes providing in situ hybridization signals significantly higher than background levels was 414. However, by mapping these probes to a recent version of the mouse genome annotation (UCSC know genes, version mm9), we realized that only 99 of them are not yet associated with protein-coding genes. Only three probes were characterized by a Pearson correlation coefficient >0.8. However, it was very interesting to notice that one of these probes corresponded to the 2900097C17Rik gene, which has been previously identified as strongly enriched in dendrites by human expert-based inspection of ABA [43]. In consideration of the high specificity of this result and of the small number of non-coding RNAs with very high score, we decided to better analyze the other 6 genes characterized by a Pearson correlation coefficient of at least 0.7 (Table 3). We hypothesized that if the RNA encoded by these genes is actually transported in the mouse hippocampal neuropil, it should be enriched in the synaptodendrosomal compartment, as it turns out to be the case for mRNAs encoding proteins of synaptic relevance, for ncRNA or for microRNAs [32], [44]–[46]. Therefore, we tested this hypothesis by analyzing the relative abundance of the corresponding RNA in crude synaptosomes versus total brain, by means of qRT-PCR. As expected, the RNA of the 2900097C17Rik gene is present at significant level in the synaptosomal compartment, comparable to the one of Fmr1 mRNA, that is located at the synapse [47] and whose synaptosome/total brain ratio is equal to 1 (not shown). Conversely, we did not detect significant expression for the 8030498B09Rik, LOC433089 and LOC435897 genes, and we detected low RNA levels for the 2700046G09Rik gene, although in this case the synaptosomal enrichment was comparable to the positive control (Table 3). The A830039N20Rik and TC1430156 RNAs were detected at levels significantly higher than background (Table 3). However, even the TC1430156 RNA was most likely a false positive results, because it did not show significant synaptosomal enrichment and because it corresponds to the sequence of Rian, an imprinted RNA accumulated in nucleus [48]. In contrast, the A830039N20Rik gene represents a truly positive result, because its synaptosomal enrichment was even higher than the positive control (Table 3). Accordingly, visual inspection of the ABA sections revealed for these gene a clear granular positivity in the proximal neuropil of pyramidal cells, which is qualitatively comparable to the positivity displayed by the 2900097C17Rik, although it is quantitatively less intense (Fig. 5).

**Table 3:** Relative expression in adult brain and synaptosomes of the top-scoring non-coding RNAs identified by the Hippo-ATESC pipeline, as determined by qRT-PCR.

Gene Name	ABA	Entrez gene ID	Pearson	Synaptosomal levels(A.U)	Total brainlevels (A.U.)	Ratio
8030498B09Rik	70227944	77547	0.873	ND	ND	ND
2900097C17Rik	71764607	347740	0.856	16.72±0.49	17.21±1.6	0.97
LOC433089	71789951	433089	0.831	ND	ND	ND
A830039N20Rik	69514374	268723	0.776	5.23±0.07	3.83±0.3	1.37
2700046G09Rik	69202980	67188	0.764	0.06±0.01	0.06±0.01	0.93
LOC435897	71022615	435897	0.744	ND	ND	ND
TC1430156 (Rian)	74580805	75745	0.708	8.46±0.7	13.71±0.82	0.62

In the last column is reported the ratio between synaptosomal and total brain levels. A.U. = arbitrary units.

### Conclusions

The Hippo-ATESC pipeline displayed a high exploratory ability in the recognition of neuropil-encoded genes from high resolution ABA images. This method can be seen as a data mining tool that can give helpful information to select target genes whose nature will be studied and confirmed using biological tests. Therefore, the list of candidate neuropil-enriched protein-coding genes that we have provided could represent an important resource for the detection of new genes involved in synaptic plasticity. Indeed, by validating some of the results we have identified a new bona-fide ncRNA enriched in synaptosomes. It will be very interesting to address the functional role of this molecule, by altering its expression levels in neuronal cells.

## Supporting Information

Table S1 List of the subset of features used for model training. Notes: “Energy2” stands for second order feature Energy. The number in brackets represents the size of the window on which the feature was calculated, when more than one window size was used for the region under consideration.
(PDF)

Table S2 List of the probes associated to protein-coding genes characterized by a Pearson coefficient of 0.8 or more. Gene names marked by a star correspond to those mapping to the mitochondrial genome. ABA indicates the Allen Brain Atlas experiment number. In the last three columns the gene is marked if it has been identified in the respective study.
(PDF)
