# Supplementary material for: Visual Search of Neuropil-Enriched RNAs from Brain In Situ Hybridization Data through the Image Analysis Pipeline Hippo-ATESC
Source: PLoS One. 2013 Sep 9;8(9):e74481. doi: 10.1371/journal.pone.0074481 (PMC3767670; doi:10.1371/journal.pone.0074481)
Supplement: Table S1 — List of the subset of features used for model training. Notes: “Energy2” stands for second order feature Energy. The number in brackets represents the size of the window on which the feature was calculated, when more than one window size was used for the region under consideration. (PDF) [file pone.0074481.s001.pdf]

Supplemental Table S1

1)	CA1 C	Mean
2)	CA1 C	StdDev
3)	CA1 C	Skewness
4)	CA1 C	Kurtosis
5)	CA1 C	Energy
6)	CA1 C	Contrast
7)	CA1 C	Correlation
8)	CA1 C	Energy2
9)	CA1 C	Homogeneity
10)	CA1 B	Mean
11)	CA1 B	Skewness
12)	CA1 B	Kurtosis
13)	CA1 B	Energy
14)	CA1 B	Contrast
15)	CA1 B	Correlation
16)	CA1 B	Energy2
17)	CA1 B	Homogeneity
18)	CA1 D	Mean
19)	CA1 D	StdDev
20)	CA1 D	Entropy (14)
21)	CA1 D	Contrast (14)
22)	CA1 D	Energy2 (14)
23)	CA1 D	Homogeneity (14)
24)	CA1 D	Energy (28)
25)	CA1 D	Contrast (28)
26)	CA1 D	Homogeneity (28)
27)	CA1 A	Mean (18)
28)	CA1 A	Energy (18)
29)	CA1 A	Entropy (18)
30)	CA1 A	Correlation (18)
31)	CA1 A	Energy2 (18)
32)	CA1 A	Homogeneity (18)
33)	CA1 A	StdDev (36)
34)	CA1 A	Energy (36)
35)	CA1 A	Entropy (36)

36)	CA1 A	Correlation (36)
37)	CA1 A	Energy2 (36)
38)	CA3 B	StdDev
39)	CA3 B	Contrast
40)	CA3 B	Homogeneity
41)	CA3 D	Energy2 (28)
42)	CA3 A	Correlation (18)
43)	DG C	Kurtosis
44)	DG B	StdDev
45)	DG B	cv
46)	DG B	Entropy
47)	DG B	Contrast
48)	DG B	Correlation
49)	DG E	Mean (18)
50)	DG E	Mean (36)
51)	DG A	Correlation (18)
52)	DG A	Correlation (36)

List of the subset of features used for model training. Notes: “Energy2” stands for second order feature Energy. The number in brackets represents the size of the window on which the feature was calculated, when more than one window size was used for the region under consideration.
