# Supplementary material for: Visual Search of Neuropil-Enriched RNAs from Brain In Situ Hybridization Data through the Image Analysis Pipeline Hippo-ATESC
Source: PLoS One. 2013 Sep 9;8(9):e74481. doi: 10.1371/journal.pone.0074481 (PMC3767670; doi:10.1371/journal.pone.0074481)
Supplement: Table S2 — List of the probes associated to protein-coding genes characterized by a Pearson coefficient of 0.8 or more. Gene names marked by a star correspond to those mapping to the mitochondrial genome. ABA indicates the Allen Brain Atlas experiment number. In the last three columns the gene is marked if it has been identified in the respective study. (PDF) [file pone.0074481.s002.pdf]

Table S2: *List of the probes associated to protein-coding genes characterized by a Pearson coefficient of 0,8 or more. Gene names marked by a star correspond to those mapping to the mitochondrial genome. ABA indicates the Allen Brain Atlas experiment number. In the last three columns the gene is marked if it has been identified in the respective study.*

Gene Name	ABA	Entrez gene ID	Pearson	Lein, 2007	Cajigas, 2012	Zhong, 2006	Poon, 2006
Ddn	71212512	13199	0,964531	X	X		
9530085C10Rik*	73906215	N/A	0,961223				
Camk2a	79360274	12322	0,960742	X	X		X
0610005I03Rik*	74357749	N/A	0,956784				
Psd	69352896	73728	0,95432	X	X		
Uqcrb	293241	67530	0,954282				
Mtap2	69549641	17756	0,954234	X	X		
0610009I12Rik*	74357773	N/A	0,95398				
0610006F12Rik*	74357761	N/A	0,949916				
Git1	69672880	216963	0,947688		X		
Slc25a23	71280844	66972	0,945908				
Rnf10	294052	50849	0,945118	X	X		
Muc10	72737	17830	0,943135				
Rpl23	70813131	65019	0,938613				
7420498E04Rik*	73927706	N/A	0,938129				
Senp2	227749	75826	0,935844		X		
Bcap29	72739	12033	0,931886				
Mt1	67767450	17748	0,930348				
Ppp1r9b	68151446	217124	0,92951	X	X	X	
Tubb2a	69838608	22151	0,927922		X		
Tnfaip6	513012	21930	0,926779				
Paf1	248542	54624	0,926624				
Rpl11	199144	67025	0,926277				
Bxdc1	248538	67239	0,925449				
Anapc5	69838210	59008	0,92315				X
Eef1a1	632492	13627	0,921128	X	X		X
AL033314	293901	56463	0,921123				
LOC328602	74579467	328602	0,919035				
Rnf122	199225	68867	0,918291				
0610005G16Rik*	74357755	N/A	0,917025				
Slc9a6	69874078	236794	0,915332				
Acsl5	70300174	433256	0,914563				
0610008A24Rik*	74357767	N/A	0,912877				
Plp1	75496529	18823	0,912137				
Tsg101	293262	22088	0,911081				
Bzw1	293900	66882	0,911074				
Mdm4	512777	17248	0,907725				
Apoe	67936406	11816	0,904798				
4931406I20Rik	70528073	66743	0,901962				
Ixl	199204	67224	0,90135				
2900097C17Rik	71764607	347740	0,900709				
Ccni	70445658	12453	0,898847		X	X	
Mmachc	70201588	67096	0,898234				
Aldoc	275696	11676	0,895739				
Nelf	74821712	56876	0,895636		X	X	
Nadk	532589	192185	0,895446				
Eif3s4	293259	53356	0,895206				
Asah1	413772	11886	0,892541				
Spsb4	248553	211949	0,892311				
Fxn	69672575	14297	0,891006				
Zfp365	70200776	216049	0,890939				
Neu1	532607	18010	0,890572				
Hagh	532605	14651	0,890081				
Rad51	248544	19361	0,889634	X			
8430410A17Rik	199201	232210	0,889188				
Oxct1	72720	67041	0,889147				
Hsbp1	72734	68196	0,888394				
2610017I09Rik	74635596	66297	0,887831				
Scotin	512998	66940	0,88779				
Plp1	75457515	18823	0,887758				
A530057D15Rik*	73949707	N/A	0,887638				
Tmsb4x	512993	19241	0,886634	X			
Ndufa2	512999	17991	0,885292				
Ppgb	512767	19025	0,884338				
Rpl4	69095958	67891	0,882776		X	X	X
Hint3	199218	66847	0,881541				
Spag9	70302246	70834	0,880958				
Rpl6	69818317	19988	0,880711		X		
4921517L17Rik	72712	70873	0,879447				
Chd6	70812978	71389	0,8779				
Man1a	75457370	17155	0,877889				

Cttnbp2nl	532609	80281	0,877757				
Cst3	75492862	13010	0,87641			X	
AW049604	69059974	106014	0,876397				
Pias2	294017	17344	0,876373				
Tuba1	68844164	22142	0,876186		X		
Ndufa13	69838573	67184	0,876138				
Plp1	75492744	18823	0,875936				
Dohh	413812	102115	0,875905				
Alas1	199198	11655	0,875897				
3110001D03Rik	67815953	66928	0,875237				
Eef2	323166	13629	0,875005		X		X
Nbn	69262299	27354	0,874627				
Nudt2	199220	66401	0,874318				
Toe1	532669	68276	0,873023				
Mus81	199202	71711	0,872929				
Cst3	75496215	13010	0,872613			X	
Cst3	75496212	13010	0,872129			X	
Cst3	75496720	13010	0,872113			X	
Cdk9	293258	107951	0,870548			X	
Dlgh4	68193007	13385	0,870049	X	X		
Neu3	74003385	50877	0,870002				
Atxn7l3	71764425	217218	0,869629		X		
Clta	69838610	12757	0,86846				
Dok1	68910956	13448	0,86784				
BC005537	275692	79555	0,867217				
Tor1aip1	512990	208263	0,866613				
Fliih	68193045	14248	0,866368				
Pygb	227737	110078	0,865982				
Dlgap1	70528599	224997	0,865508				
Cand1	69608420	71902	0,864985				
Cst3	75457610	13010	0,863627			X	
Glul	67978757	14645	0,863163				
Atp6v1f	393251	66144	0,861925		X		
Ctbp1	69059817	13016	0,861113				
Taok1	70527993	216965	0,860877		X		
1700020C11Rik	556992	67900	0,860828				
Cst3	75496211	13010	0,860516			X	
3100002L24Rik	248541	66376	0,85759				
Gpm6b	74357593	14758	0,857046		X		
Psip1	70565135	101739	0,85609				
Cst3	75457611	13010	0,855961			X	
Polr3h	393250	78929	0,85531				
Cst3	69117385	13010	0,85506			X	
Ppp2r2c	69817240	269643	0,854493				
Psmd2	68157415	21762	0,854109		X		
Crtc1	69524515	382056	0,85353		X		
Cst3	75496213	13010	0,853456			X	
Cst3	75492852	13010	0,852923			X	
Cyc1	323186	66445	0,852549				
Cst3	75496721	13010	0,852126			X	
TC1498646	70812990	N/A	0,851414				
Nap1l2	71234531	17954	0,851068		X		
Rab11b	69816739	19326	0,850917				
Cst3	75496731	13010	0,85052			X	
Tmem49	532588	75909	0,850509				
Cst3	75492864	13010	0,850508			X	
Cst3	75492865	13010	0,850239			X	
Osgep	413823	66246	0,849673				
Vps35	293904	65114	0,848999		X		
4930470H14Rik	71891749	75815	0,848486				
Lasp1	512991	16796	0,848235				
Dynll2	564758	68097	0,848166				
Ndufb3	70473927	66495	0,847097				
Ahcyl1	69838759	229709	0,846379				
Sbk1	532706	104175	0,845566	X			
Flot1	72735	14251	0,845016		X		
Sept9	69262288	53860	0,844384				
TC1515580	70813173	N/A	0,844282				
Mt3	69117074	17751	0,843511				
Fbxo22	69015482	71999	0,84336				
Dync2li1	378552	213575	0,842911				
Scg3	71608205	20255	0,842879		X	X	
Angel2	67767464	52477	0,842689				
Copz2	199224	56358	0,842664				
Psd3	69095967	234353	0,84238		X		
1810008A14Rik	199214	66268	0,842254				
4833439L19Rik	378550	97820	0,842063				
Centg1	73513639	216439	0,841557				

Tm9sf3	413788	107358	0,841368				
Cabp7	70305592	192650	0,840325		X		
Jph3	69735670	57340	0,840293		X		
LOC545133	69262305	545133	0,839122				
Plp1	75457512	18823	0,838513				
TC1568600	70919872	N/A	0,838461				
Uba52	69013702	22186	0,838097		X	X	
Smad7	69290501	17131	0,83798				
Chd3	385790	216848	0,837866				
Bre	73818810	107976	0,836893		X		
Gpr158	73497162	241263	0,836768		X		
Prrg2	71608228	65116	0,836474				
Atp5b	70528264	11947	0,836164	X	X		
Map2k4	227736	26398	0,836099		X		
Mras	71764156	17532	0,835638		X		
Prep	69059821	19072	0,834941		X		
Nr2c2	70593328	22026	0,834857				
Pea15	68193012	18611	0,834695				
Sec14l2	71325450	67815	0,834018				
Rpl13	69028599	270106	0,833388				
Fbxo31	69261394	76454	0,832416		X		
Rasa3	69817970	19414	0,832186				
Sparcl1	70429447	13602	0,832126				
Mvp	275693	78388	0,830896				
Zfp84	293893	74352	0,830222				
Serpine2	70429429	20720	0,829305				
Tomm34	532647	67145	0,829218				
5730422E09Rik	70787191	433966	0,829061				
Cdc42se2	70527981	72729	0,828898				
Ppp5c	413805	19060	0,828547		X		
Tbccd1	70305060	70573	0,828542				
Mm125511	72182975	N/A	0,827797				
D7Wsu128e	70431265	28018	0,827462		X		
Scd3	69873427	30049	0,827376				
Rab7	532633	19349	0,827239				
Aph1c	69352730	68318	0,82697				
Sh3rf1	70303624	59009	0,826143		X		
Hdac11	69080509	232232	0,825407				
Mapk8ip1	69752059	19099	0,824548			X	
Copz1	385996	56447	0,824176				
Atpaf1	69262300	230649	0,824137				
Freq	69256652	14299	0,823974		X		
Narf	583563	67608	0,823859				
Taok1	68341645	216965	0,822997		X		
Slc30a9	73573181	109108	0,822678		X		
Clic1	86352	114584	0,821008				
2410091C18Rik	70527963	73694	0,820697				
Psmf1	532650	228769	0,820171				
2600011E07Rik	74821568	72125	0,819624				
Lrp3	71022633	435965	0,818753		X		
Exdl2	70430726	97827	0,81844		X		
Scd2	68633119	20250	0,818285				
Dab2ip	71496405	69601	0,817773				
Trim26	70565345	22670	0,817337				
Tmem38a	71612429	74166	0,816712		X		
Snph	69885582	241727	0,816351				
9030425E11Rik	70445538	71566	0,816289				
Spin	70429453	20729	0,81618				
4631402N15Rik	70303833	70793	0,815192				
Slc9a3r1	69874066	26941	0,81511				
Pigq	294046	14755	0,814524				
Tuba2	77465048	22143	0,81427				
Lypla1	70546099	18777	0,814199				
Clstn3	69013572	232370	0,813973				
Sar1b	69873397	66397	0,813634				
Sos2	71735062	20663	0,813458				
Rab11fip4	71378475	268451	0,813062		X		
Pabpc1	71616794	18458	0,813005		X		X
Arf5	69526791	11844	0,812915		X		
St13	532638	70356	0,812755				
Clu	77800883	12759	0,812476				
Rab15	69080008	104886	0,812012		X		
Rpl13a	71764512	22121	0,811856		X		
Sec14l1	70429423	74136	0,811585		X		
Eif3s3	67765724	68135	0,810766				
Atxn2	69873421	20239	0,810265				
Phf14	199216	75725	0,809414				
1200011O22Rik	69838515	71735	0,809409				

Cox4i1	69568980	12857	0,809044				
Zfp366	70200782	238803	0,808605				
Lrpprc	70546087	72416	0,808377				
LOC434058	70787227	434058	0,808341				
Pip5k2c	68445907	117150	0,807883		X		
Plp1	75457514	18823	0,807386				
Pdha1	293257	18597	0,807329				
E330014M11Rik*	74076114	N/A	0,807128				
Arl6ip2	513010	56298	0,807072				
Brd2	70305496	14312	0,806556				
BC026590	69529017	230234	0,806183				
Chchd3	176776	66075	0,805757				
Aip	69526456	11632	0,80569				
Azi2	512987	27215	0,805018				
Vgf	70299935	381677	0,804772		X		
Plp1	75496147	18823	0,80465				
Tnrc5	70429483	72029	0,80425		X		
Hkr2	199200	232878	0,804168				
Sub1	71608391	20024	0,804089				
Trappc6b	68637862	78232	0,80397		X		
Serinc3	71616360	26943	0,803847				
Tpm3	199223	59069	0,802984				
Zfp553	70539192	233887	0,802235				
Tpd52l2	413802	66314	0,802093				
Stac2	74357598	217154	0,801418				
Spon1	69059948	233744	0,800582		X		
Sparc	70429441	20692	0,800252				
Prkcbp1	71833000	228880	0,800184				
D10Ertd641e	199227	52717	0,800144				
Prpf6	119683	68879	0,800133				
